# Visceral adiposity index outperforms conventional anthropometric assessments as predictor of diabetes mellitus in elderly Chinese: a population-based study

**DOI:** 10.1186/s12986-021-00608-6

**Published:** 2021-09-25

**Authors:** Meng-Ting Tsou, Yu-Chen Chang, Ching-Ping Hsu, Yang-Che Kuo, Chun-Ho Yun, Wei-Hsin Huang, Kuang-Chun Hu, Chia-Yuan Liu, Ying-Ju Chen, Kuo-Tzu Sung, Chuan-Chuan Liu, Chung-Lieh Hung, Jen-Yuan Kuo, Tung-Ying Chen, Ta-Chuan Hung, Hung-I. Yeh

**Affiliations:** 1 Department of Family Medicine, MacKay Memorial Hospital, Taipei City, 10449 Taiwan, ROC; 2 Department of Occupation Medicine, MacKay Memorial Hospital, Taipei City, 10449 Taiwan, ROC; 3 Department of Health Evaluation Center, MacKay Memorial Hospital, New Taipei City, 25245 Taiwan, ROC; 4 Department of Health Evaluation Center, MacKay Memorial Hospital, Taipei City, 10449 Taiwan, ROC; 5 Department of Radiology, MacKay Memorial Hospital, Taipei, Taiwan, ROC; 6 Department of Medicine, MacKay Medical College, New Taipei City, 25245 Taiwan, ROC; 7 MacMacKay Junior College of Medicine, Nursing, and Management, Taipei City, 11260 Taiwan, ROC; 8 Division of Gastroenterology, Department of Internal Medicine, MacKay Memorial Hospital, Taipei City, 10449 Taiwan, ROC; 9 Department of Telehealth, MacKay Memorial Hospital, New Taipei City, Taiwan, ROC; 10 Cardiovascular Division, Department of Internal Medicine, MacKay Memorial Hospital, No. 92, Sec. 2, Zhongshan N. Rd., Taipei City, 10449 Taiwan, ROC; 11 Department of Pathology, MacKay Memorial Hospital, 92, Sec 2, Chung Shan North Road, Taipei, 10449 Taiwan, ROC

**Keywords:** Body mass index, Body shape index, Diabetes mellitus, Obesity, Visceral adiposity index, Waist circumference

## Abstract

**Background:**

This study assessed the performance of visceral adiposity index and body shape index in predicting diabetes mellitus (DM) risk and compared their predictive ability to that of body mass index and waist circumference.

**Methods:**

Among 8249 consecutive subjects who attended the Nationwide Health Check Up System for Senior Citizens (≥ 65 years) between 2008 and 2018, we examined the associations of several adiposity indices with DM risk and explored gender differences.

**Results:**

Among all adiposity indicators, Chinese visceral adiposity index (CVAI) demonstrated the highest discriminatory ability for diabetes mellitus with area under receiver operating characteristic curves (AUC) of 0.65, 0.68, and 0.66 for men, women, and all participants, respectively, and optimal cut-offs set as 126.09 in men and 117.77 in women. Compared with body shape index (ABSI), both CVAI and VAI were strongly associated with baseline DM (adjusted OR: 4.85, 95% CI: 4.05–5.82 and 4.22, 95% CI: 3.53–5.05 for 4th vs 1st quartile groups by CVAI and VAI, *P* < 0.001), which was more pronounced in older adult women (*P*_interaction_ < 0.05). Over a median of 5.25 years (IQR: 3.07–6.44 years) follow-up, Cox regression models showed higher predictive ability of CVAI and VAI compared to ABSI. Further, both CVAI and VAI independently predicted new-onset DM (adjusted HR: 1.29, 95% CI: 1.22–1.37 and 1.16, 95% CI: 1.11–1.21 by CVAI and VAI) and composite endpoint of new DM and death among those without baseline DM.

**Conclusions:**

Our population-based data demonstrated that Chinese visceral adiposity index may serve as a superior clinical indicator of diabetes when compared with conventional anthropometric indices among older adult Chinese, especially in women.

**Supplementary Information:**

The online version contains supplementary material available at 10.1186/s12986-021-00608-6.

## Introduction

A higher prevalence of diabetes mellitus in the older adult population is a critical public health issue worldwide [1]. With a prevalence that is particularly high in older adult populations, diabetes mellitus can cause systemic organ damage and lead to cardiovascular diseases. Diabetes in older adult populations can result due to genetic background and/or longer life expectancy that leads to decreased insulin secretion or higher insulin resistance, which can result from multiple factors such as central obesity or metabolic syndrome mediated by excessive visceral adipose tissue (VAT) accumulation [2, 3]. Aging may also trigger adverse pro-inflammatory cytokine secretions through visceral fat redistribution, leading to metabolic disorders, such as diabetes mellitus [4]. Previous studies have shown that age, gender, and ethnicity are the main determinants of body fat distribution [5]. Due to a shift of adiposity from the peripheral to a more central truncal area, visceral fat increases over 200% in men and 400% in women between the 3rd and 7th decades, respectively [5].

Studies have demonstrated that Asian populations are at a higher risk for metabolic cardiovascular diseases (such as diabetes), even when compared to whites with a higher BMI and similar baseline characteristics [6, 7], leading to researchers revisiting the BMI threshold in defining obesity for Asians [8]. Central obesity, especially abdominal VAT, has been proposed to contribute to metabolic and diabetes risk beyond [9]. Recent studies have shown that individuals of Japanese descent living in Western societies differ in body fat mass and body fat distribution when compared to whites independent of BMI [10]. The association of VAT with diabetes mellitus also appears to be stronger in South Asian men than in European men and even more so in women [11], suggesting a higher clinical impact of VAT on diabetes mellitus in South Asian women [9]. Furthermore, when compared with white women at similar BMI levels, Asian women can have greater abdominal and visceral adiposity and be prone to a higher risk of metabolic- or obesity-related diseases [4].

By utilizing echocardiography, magnetic resonance imaging (MRI) and computed tomography (CT) have confirmed that visceral adiposity is a valuable indicator of insulin resistance, irrespective of body mass [12, 13]. Despite these advances, using imaged-based assessment of visceral adiposity was largely limited due to its higher costs and associated technical challenges. The Chinese visceral adiposity index (CVAI), which is estimated based on age, BMI, waist circumference (WC), total triglycerides (TG), and high-density lipoprotein cholesterol (HDL-C), was developed to assess the presence of DM in the Chinese population [14]. However, validation of this measure using imaging-based VAT measures and the potential association of CVAI with diabetic risk in older Asian populations remains largely unexplored. Alternatively, a Body Shape Index (ABSI) uses WC and BMI to predict fat distribution [15, 16] and has been shown to be a reliable index of body fat accumulation, yet there is little data on its application in the older adult population [17].

Our study hypothesizes that noninvasive surrogate markers that can be helpful in assessing body fat distribution and in the prediction of diabetes mellitus risk. We examined the associations between CVAI, ABSI, and diabetes mellitus risk and investigated their ability to identify diabetes mellitus when compared with BMI and WC alone in the an older adult population in Taiwan.

## Materials and methods

### Study design and population

This population-based study comprised 8,500 consecutive subjects who attended the Nationwide Health Check Up System for Senior Citizens between 2008 and 2018 at the Health Evaluation Center in Mackay Memorial Hospital, a tertiary teaching center. Detailed information including medical histories for chronic illnesses, structured questionnaires for personal habits, physical examination including systolic and diastolic blood pressures, anthropometric measurements, and biochemical marker levels were obtained in all subjects participating in this program. Those who had end-stage renal disease, Stage 5 (estimated glomerular filtration rate (eGFR) < 15, ml/min/1.73 m^2^), or those undergoing renal replacement therapy were excluded (n = 146). We also excluded participant with a history of cancer (n = 128). History of cardiovascular disease (CVD) was defined as previous myocardial infarction, coronary artery disease (including elective intervention), cerebrovascular events, prior hospitalization for congestive heart failure, and peripheral arterial disease. The presence of hypertension (HTN) was defined as a previous diagnosis of disease or current medication use for HTN. Venous samples were collected by blood tests from all patients, followed by a detailed physical examination taken by the family physician. After applying exclusion criteria, data from 8249 subjects were analyzed. Diabetes mellitus was defined as a fasting plasma glucose (FPG) ≥ 7.0 mmol/L, a previous diagnosis of diabetes mellitus (data was obtained from structured questionnaire responses or from the information stored in the electronic medical record), or current use of anti-diabetic medications. Among 8249 study subjects, 1539 (18.7%) had type 2 DM (stage 3 and 4, definition by dysglycemia-based chronic disease [DBCD]) [14]. Subjects with suspected hyperglycemia (based on their fasting glucose level), though without a confirmed diagnosis of diabetes mellitus, were referred for further checkups according to the current guideline recommendations [18]. This study was approved by the ethical institutional committee of Mackay Memorial Hospital (IRB No: 18MMHIS137) for retrospective data analysis and did not require informed consent of study participants. All data were fully anonymized.

### Anthropometric measurements

Body weight (kg) and height (m) were measured according to standard methods. WC was measured at the middle point between the bottom of the ribcage and the uppermost border of the iliac crest at the end of exhalation with the patient in a standing position. Trained nurses used standard mercury sphygmomanometers to measure blood pressure two consecutive times at 3–5 min intervals during one visit.

### Laboratory biochemical information

Sample collection and analysis were performed in a standard laboratory with international accreditation (ISO-15189). All subjects were requested to fast for more than 8 h before venous blood sampling; samples were collected in a BD Vacutainer SSTTM (Becton Dickinson, Franklin Lakes, NJ, USA) sample collection tube. Sample collection and analysis principles were based on the standard requirements given in the Clinical Laboratory Standards Institute guidelines (Specimen Choice, Collection, and Handling; Approved Guideline H18-A3). FPG (via a hexokinase method); lipids including total cholesterol (TC), triglycerides (TG), low-density lipoprotein cholesterol (LDL-C), and high-density lipoprotein cholesterol (HDL-C); as well as uric acid (UA) were measured using a biochemical auto-analyzer (A Hitachi 7170 automatic analyzer; Hitachi Corp., Hitachinaka, Ibaraki, Japan). Quality control and instrument operation were done in accordance with standard procedures dictated by the guidelines of the Clinical Laboratory Standards Institute. Samples were assessed in triplicate, and the final values (after quality control) were confirmed to be in the linear range using an internal standard.

### Assessment of adiposity

The VAI score was calculated using the specific formula developed for the Chinese population [19]:

- Men: CVAI =  − 267.93 + 0.68 × Age + 0.03 × BMI + 4.00 × WC + 22.00 × log10 (TG) − 16.32 × HDL;
- Women: CVAI =  − 187.32 + 1.71 × Age + 4.23 × BMI + 1.12 × WC + 39.76 × log10 (TG) − 11.66 × HDL.
- ABSI was calculated as WC/(BMI^2/3^ × height^1/2^) and expressed in m^11/6^ kg^−2/3^ [16].
- BMI was calculated as weight/height squared (kg/m^2^).
- VAI was derived using the following formula [20]
  $$\begin{aligned} & {\text{VAI}} = \frac{{{\text{WC}}}}{{39.68 + (1.88 \times {\text{BMI}})}} \times \left( {\frac{{{\text{TG}}}}{1.03}} \right) \times \left( {\frac{1.31}{{{\text{HDL}} - {\text{C}}}}} \right) \\ & {\text{VAI}} = \frac{{{\text{WC}}}}{{36.58 + (1.89 \times {\text{BMI}})}} \times \left( {\frac{{{\text{TG}}}}{0.81}} \right) \times \left( {\frac{1.52}{{{\text{HDL}} - {\text{C}}}}} \right) \\ \end{aligned}$$

New-onset diabetes was defined as newly diagnosed diabetes, after the baseline study indexed date, in non-diabetes mellitus study participants (n = 6710) using the same clinical guidelines as used for assessing baseline diabetes mellitus. Considering that new-onset diabetes mellitus can be confounded by the high mortality rate before the development of diabetes mellitus in the older adult population, the prognostic implications of CVAI/ABSI on a composite endpoint of incident diabetes mellitus/mortality were examined.

### Statistical analysis

Descriptive analyses were presented according to gender-specific quartiles of CVAI and ABSI scores to control for the well-known sexual dimorphism in body composition. The results are presented as mean with standard deviation (SD) and median with interquartile range (IQR: 25%–75%; given in parentheses) for normalized and skewed continuous variables, respectively. Categorical variables are expressed as numbers and percentages. Baseline continuous demographic information across CVAI/ABSI quartiles were compared using linear regression for trend test (as p for trend). Chi-squared test was performed to assess differences in proportions across groups.

Sex-stratified correlations between CVAI/ABSI and three anthropometric indices and metabolic parameters were evaluated. Multivariable logistic regression models were performed to estimate the risk of diabetes mellitus (presented as ORs and 95% CI) in relation to these indices in the three models for men and women, respectively. Confounders were chosen from prior literature report [21]. Receiver operating characteristic (ROC) curve analyses was used to compare the discriminative performance of CVAI, VAI and ABSI compared with BMI and WC for assessing underlying diabetes mellitus risk. A user-written command was used to calculate the Youden index for optimal clinical cut-offs of CVAI, VAI and ABSI in identifying the presence of baseline type 2 diabetes mellitus [19]. By defining the standardized unit shift of baseline age, anthropometric measures, and adiposity measures (CVAI, VAI and ABSI), we explored the relationships among these indices with baseline diabetes mellitus risk. Cox proportional hazard regression models were constructed to explore the predictive values of CVAI/VAI/ABSI in predicting new-onset diabetes mellitus among the remaining baseline non-diabetes mellitus study participants (n = 6710), with differences in gender tested by interaction analysis (CVAI/VAI or ABSI tertiles treated as ordinal, continuous variable).

All statistical analyses were conducted using SAS software (version 9.4, SAS, Cary, NC, USA). A two-tailed statistical measure was used, with a *P* < 0.05 considered significant.

## Results

### Baseline characteristics of the study population

The mean age among the 8249 study participants was 74.1 ± 7.1, and 56.4% (n = 4649) of the subjects were women (Table 1). In both genders significant dose–response relationships were observed between higher CVAI and advanced age, larger anthropometric indices (i.e., BMI, WC), increased blood pressure, increased fasting glucose, elevated UA level, more unfavorable HDL-C/TG profiles, and worsened renal function (*P* < 0.001). Men with higher levels of CVAI were less likely to have regular exercise (both *P* < 0.05). Those with higher CVAI were more likely to have prevalent diabetes and receiving pharmacological treatment for hyperlipidemia, which probably leads to lower TC and LDL-C levels (all *P* < 0.05). Similarly, in both men and women, those with higher ABSI scores presented with higher BMI and WC, higher fasting glucose, more unfavorable HDL/TG ratio, higher UA levels, and higher CVAI scores (all *P* < 0.001). Additionally, in both men and women, the proportion of participants who had regular exercise and diabetes decreased with increasing ABSI scores (both *P* < 0.001) (Table 1). Overall, women had significantly higher CVAI scores (118.4 ± 33.8 vs 111.0 ± 41.8) yet slightly lower ABSI score (0.12 ± 0.01 vs 0.13 ± 0.01, both *P* < 0.001) compared with men.

**Table 1:** Demographic and clinical characteristics of study participants (n = 8249) in Taiwan. a. Across CVAI quartiles; b. Across ABSI quartiles

	Men (n = 3600)					Women (n = 4649)				
	Q1 (< 84.47)	Q2 (84.47–111.45)	Q3 (111.45–138.01)	Q4 (≥ 138.01)		Q1 (< 94.66)	Q2 (94.66–117.87)	Q3 (117.87–141.57)	Q4 (≥ 141.57)	
	n = 900	n = 900	n = 900	n = 900	*P*	n = 1162	n = 1163	n = 1162	n = 1162	*P*
*a. CVAI quartiles*										
Age, years	74.55 (7.00)	74.31 (7.06)	74.67 (7.45)	75.30 (7.25)	0.025	70.20 (5.26)	72.32 (6.14)	74.68 (6.79)	77.40 (7.09)	< 0.001
BMI, kg/m^2^	21.22 (2.16)	23.63 (1.78)	25.23 (1.88)	27.80 (2.77)	< 0.001	20.81 (2.15)	23.23 (1.98)	25.03 (2.17)	28.15 (3.29)	< 0.001
SBP, mmHg	131.89 (19.99)	135.18 (18.90)	135.79 (19.05)	137.62 (18.99)	< 0.001	131.62 (20.71)	136.22 (20.16)	139.93 (20.59)	144.63 (21.53)	< 0.001
DBP, mmHg	73.24 (12.00)	75.00 (11.09)	75.67 (11.37)	76.36 (11.51)	< 0.001	71.01 (11.37)	72.86 (11.66)	74.05 (11.30)	74.75 (11.71)	< 0.001
WC, cm	75.49 (4.92)	84.10 (2.65)	89.42 (2.79)	98.42 (5.75)	< 0.001	71.90 (5.82)	78.60 (5.49)	84.07 (5.73)	92.77 (8.93)	< 0.001
HbA1c, %	5.89 (0.87)	6.02 (0.88)	6.39 (1.30)	6.59 (1.34)	< 0.001	5.83 (0.83)	6.03 (0.78)	6.48 (1.22)	6.74 (1.27)	< 0.001
FPG, mmol/l	5.62 (1.03)	5.80 (1.09)	5.94 (1.15)	6.34 (1.68)	< 0.001	5.47 (0.86)	5.71 (1.06)	6.01 (1.50)	6.40 (1.84)	< 0.001
TC, mmol/l	4.92 (0.88)	4.84 (0.91)	4.86 (0.95)	4.68 (0.87)	< 0.001	5.37 (0.87)	5.30 (0.94)	5.20 (0.94)	5.13 (0.96)	< 0.001
TG, mmol/l	0.79 (0.61–1.05)	0.99 (0.75–1.39)	1.25 (0.90–1.71)	1.41 (1.04–1.99)	< 0.001	0.79 (0.63–1.02)	1.10 (0.86–1.47)	1.32 (1.02–1.82)	1.63 (1.23–2.24)	< 0.001
HDL-C, mmol/l	1.56 (0.39)	1.30 (0.31)	1.20 (0.27)	1.08 (0.24)	< 0.001	1.83 (0.40)	1.56 (0.36)	1.42 (0.33)	1.29 (0.31)	< 0.001
LDL-C, mmol/l	2.96 (0.79)	3.01 (0.83)	3.00 (0.86)	2.85 (0.82)	< 0.001	3.15 (0.80)	3.21 (0.85)	3.13 (0.83)	3.06 (0.87)	< 0.001
UA, mg/dl	5.79 (1.27)	6.15 (1.30)	6.43 (1.45)	6.62 (1.48)	< 0.001	4.67 (1.00)	5.08 (1.08)	5.44 (1.25)	5.88 (1.45)	< 0.001
eGFR, ml/min/1.73 m^2^	75.7 (20.1)	72.1 (19.2)	69.3 (19.1)	68.8 (20.5)	< 0.001	84.6 (20.1)	80.1 (20.4)	75.7 (22.1)	69.2 (21.8)	< 0.001
Smoking, n (%)	119 (13.22)	115 (12.78)	134 (14.89)	138 (15.33)	0.325	12 (1.03)	12 (1.03)	18 (1.55)	17 (1.46)	0.549
Drinking, n (%)	235 (26.11)	266 (29.56)	251 (27.89)	234 (26.00)	0.278	77 (6.63)	90 (7.74)	69 (5.94)	57 (4.91)	0.039
Exercise, n (%)	243 (27.00)	217 (24.11)	198 (22.00)	186 (20.67)	0.009	189 (16.27)	286 (24.59)	320 (27.54)	290 (24.96)	< 0.001
Diabetes, n (%)	103 (11.44)	141 (15.67)	188 (20.89)	290 (32.22)	< 0.001	80 (6.88)	141 (12.12)	245 (21.08)	351 (30.21)	< 0.001
Hyperlipidemia Tx	134 (14.9)	201 (22.3)	219 (24.3)	251 (27.9)	< 0.001	284 (24.4)	370 (31.8)	344 (29.6)	408 (35.1)	< 0.001
HTN, n (%)	108 (12.00)	115 (12.78)	146 (16.22)	125 (13.89)	0.053	121 (10.41)	183 (15.74)	218 (18.76)	215 (18.50)	< 0.001
CVD, n (%)	105 (11.67)	131 (14.56)	163 (18.11)	186 (20.67)	< 0.001	84 (7.23)	140 (12.04)	178 (15.32)	259 (22.29)	< 0.001
ABSI	0.126 (0.008)	0.131 (0.007)	0.134 (0.008)	0.138 (0.008)	< 0.001	0.118 (0.017)	0.120 (0.010)	0.122 (0.010)	0.124 (0.011)	< 0.001

	Men (n = 3600)					Women (n = 4649)				
	Q1 (< 0.1261)	Q2 (0.1261–0.1322)	Q3 (0.1322–0.1379)	Q4 (≥ 0.1379)		Q1 (< 0.1137)	Q2 (0.1137–0.1199)	Q3 (0.1199–0.1267)	Q4 (≥ 0.1267)	
	n = 900	n = 900	n = 900	n = 900	*P*	n = 1162	n = 1162	n = 1163	n = 1162	*P*
*b. ABSI quartiles*										
Age, years	74.91 (7.09)	74.92 (7.15)	74.06 (6.92)	74.94 (7.58)	0.022	73.42 (6.67)	73.05 (6.58)	73.20 (6.83)	74.93 (7.34)	< 0.001
BMI, Kg/m^2^	24.12 (3.31)	24.64 (3.04)	24.67 (3.34)	24.46 (3.23)	0.001	24.64 (3.83)	24.39 (3.56)	24.21 (3.39)	23.98 (3.72)	< 0.001
SBP, mmHg	134.90 (19.90)	134.74 (19.22)	136.35 (19.17)	134.50 (19.04)	0.170	139.16 (22.30)	137.64 (21.54)	137.30 (20.48)	138.30 (20.77)	0.161
DBP, mmHg	74.11 (11.50)	74.48 (10.95)	76.17 (11.99)	75.50 (11.63)	< 0.001	73.33 (11.97)	73.13 (11.27)	72.71 (11.39)	73.51 (11.72)	0.380
WC, cm	79.85 (8.00)	85.56 (7.23)	88.92 (8.21)	93.11 (8.45)	< 0.001	75.14 (8.17)	79.64 (7.97)	83.18 (8.14)	89.38 (10.28)	< 0.001
HbA1c, %	6.04 (0.91)	6.23 (1.15)	6.31 (1.16)	6.50 (1.43)	< 0.001	6.12 (0.90)	6.32 (1.03)	6.40 (1.16)	6.54 (1.42)	< 0.001
FPG, mmol/l	5.76 (1.07)	5.84 (1.07)	5.97 (1.27)	6.13 (1.63)	< 0.001	5.72 (1.13)	5.90 (1.42)	5.93 (1.41)	6.04 (1.64)	< 0.001
TC, mmol/l	4.85 (0.87)	4.87 (0.92)	4.81 (0.88)	4.78 (0.94)	0.101	5.27 (0.91)	5.29 (0.93)	5.24 (0.96)	5.21 (0.93)	0.195
TG, mmol/l	0.95 (0.70–1.40)	1.10 (0.78–1.53)	1.11 (0.80–1.58)	1.16 (0.82–1.66)	< 0.001	1.06 (0.80–1.49)	1.16 (0.82–1.62)	1.20 (0.88–1.70)	1.25 (0.90–1.76)	< 0.001
HDL-C, mmol/l	1.36 (0.39)	1.31 (0.36)	1.25 (0.34)	1.22 (0.31)	< 0.001	1.60 (0.42)	1.54 (0.39)	1.49 (0.38)	1.48 (0.41)	< 0.001
LDL-C, mmol/l	2.96 (0.79)	2.98 (0.83)	2.95 (0.83)	2.94 (0.86)	0.760	3.14 (0.79)	3.16 (0.85)	3.14 (0.86)	3.11 (0.85)	0.426
UA, mg/dl	6.13 (1.36)	6.24 (1.37)	6.20 (1.39)	6.40 (1.50)	0.006	5.13 (1.26)	5.30 (1.28)	5.32 (1.27)	5.36 (1.33)	0.001
eGFR, ml/min/1.73 m^2^	71.3 (19.9)	71.3 (19.2)	72.0 (19.8)	71.1 (20.7)	0.76	77.9 (21.2)	78.1 (21.1)	76.9 (22.1)	76.7 (23.1)	0.02
Smoking, n (%)	107 (11.89)	119 (13.22)	135 (15.00)	145 (16.11)	0.050	9 (0.77)	14 (1.20)	14 (1.20)	22 (1.89)	0.114
Drinking, n (%)	211 (23.44)	253 (28.11)	244 (27.11)	278 (30.89)	0.005	70 (6.02)	74 (6.37)	72 (6.19)	77 (6.63)	0.942
Exercise, n (%)	259 (28.78)	233 (25.89)	188 (20.89)	164 (18.22)	< 0.001	334 (28.74)	250 (21.51)	227 (19.52)	274 (23.58)	< 0.001
Diabetes, n (%)	151 (16.78)	151 (16.78)	197 (21.89)	223 (24.78)	< 0.001	152 (13.08)	194 (16.70)	205 (17.63)	266 (22.89)	< 0.001
Hyperlipidemia Tx	199 (21.1)	213 (23.6)	204 (22.0)	189 (22.8)	0.6	400 (30.9)	329 (29.0)	357 (31.1)	320 (29.9)	0.67
HTN, n (%)	138 (15.33)	130 (14.44)	113 (12.56)	113 (12.56)	0.218	210 (18.07)	174 (14.97)	188 (16.17)	165 (14.20)	0.059
CVD, n (%)	137 (15.22)	155 (17.22)	132 (14.67)	161 (17.89)	0.190	179 (15.40)	158 (13.60)	157 (13.50)	167 (14.37)	0.527
CVAI	80.93 (37.22)	105.64 (33.47)	119.72 (36.68)	137.75 (37.43)	< 0.001	109.70 (33.84)	114.92 (32.36)	119.67 (31.45)	129.33 (34.19)	< 0.001

*ABSI* a body shape index; *BMI* body mass index; *CVAI* Chinese visceral adiposity index; *DBP* diastolic blood pressure; *DM* diabetes mellitus; *eGFR* estimated glomerular filtration rate; *FPG* fasting plasma glucose; *HDL-C* high density lipoprotein cholesterol; *HTN* hypertension; *Hyperlipidemia Tx* pharmacological treatment for hyperlipidemia; *LDL-C* low density lipoprotein cholesterol; *SBP* systolic blood pressure; *SD* standard deviation; *TC* total cholesterol; *TG* Triglyceride; *UA* uric acid; *WC* waist circumference

Data are mean (SD) or median (interquartile range), unless otherwise stated

### Validation of CVAI with MDCT-defined visceral adiposity

Data on MDCT-defined peri-cardiac and peri-aortic adiposity burden were available in 374 study subjects. A significantly positive linear correlation was observed between CVAI score and peri-cardiac fat (PCF) and peri-aortic fat (TAT) (r = 0.825 and 0.786 for PCF and TAT, respectively. Additional file 1: Fig. 1). However, modest correlations were observed among ABSI with PCF and TAT (r = 0.386 and 0.455 for PCF and TAT, respectively. Additional file 1: Fig. 1).

**Fig. 1:**
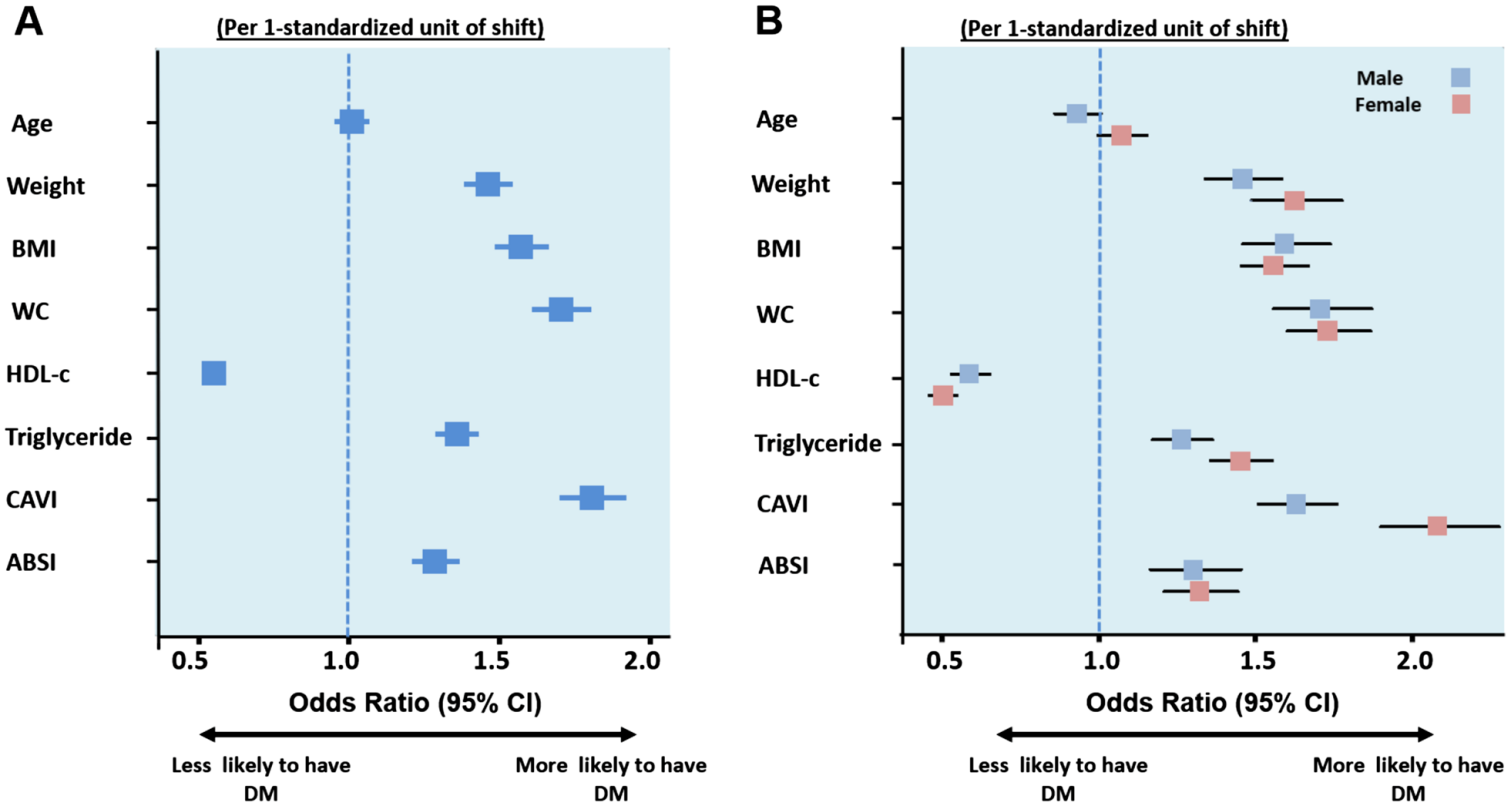
Univariate models identifying baseline diabetes risk adjusting for age, various anthropometric or VAI indices (**A**) and sex differences (**B**). *P*_interaction_ by sex < 0.05, in age, HDL, TG, and CVAI. *CVAI* Chinese visceral adiposity index; *HDL* high-density lipoprotein; *TG* triglyceride; *VAI* visceral adiposity index; *BMI* body mass index; *WC* waist circumference

### Correlation of adiposity measures with diabetes and biochemical metabolic profiles

Baseline diabetes mellitus was present in 19% (n = 1539) of all study participants. Those with baseline diabetes mellitus had significantly higher CVAI (132.4 ± 36.9 vs 111.2 ± 36.7) and ABSI (0.128 ± 0.017 vs 0.125 ± 0.011, both *P* < 0.001) scores compared with those without diabetes mellitus. Sex-stratified correlations among CVAI/ABSI and anthropometric or metabolic profiles are shown in Additional file 1: Table 1. CVAI was highly correlated with BMI (r=0.81, *P*<0.001) and WC (r=0.97, *P*<0.001) in men, and highly correlated with BMI (r=0.81, *P*<0.001) and WC (r=0.83, *P*<0.001) in women. VAI was highly correlated with TG both in men (r=0.94, *P*<0.001) and women (r=0.92, *P*<0.001). Finally, ABSI was modestly correlated with WC in men (r=0.55, *P*<0.001) and women (r=0.46, *P*<0.001), respectively.

### Association of CVAI or ABSI with diabetes risk

Unfavorable anthropometric measurements, lipid profile, and VAI (CVAI and ABSI) were all associated with higher baseline diabetes mellitus risk (Fig. 1). Additional file 1: Table 2 shows the sensitivity, specificity and corresponding optimal cut-off values of each index for identifying diabetes by gender. CVAI had the highest Youden index values for identifying diabetes in men (0.23) and in women (0.28); the optimal CVAI cut-off was 126.09 in men and 117.77 in women.

**Table 2:** Uni- and multivariate Cox models in predicting new-onset diabetes by CVAI and ABSI tertiles among study population without baseline diabetes (n = 6710)

	Unadjusted	Multivariate (Model 1)	Multivariate (Model 2)
Visceral adiposity index (CVAI)	HRs (95% CI)		
All (per 1-standard unit increment)	1.30 (1.23–1.38)***	1.30 (1.23–1.37)***	1.29 (1.22–1.37)***
CVAI Tertiles			
Q1	1 (Reference)	1 (Reference)	1 (Reference)
Q2	1.20 (1.04–1.37)*	1.19 (1.03–1.37)*	1.17 (1.02–1.35)*
Q3	1.77 (1.56–2.03)***	1.75 (1.53–2.00)***	1.73 (1.51–1.98)***
*P*_*interaction*_* for sex*	0.091	0.10	0.18
Adiposity index (VAI)	HRs (95% CI)		
All (per 1-standard unit increment)	1.16 (1.11–1.21)***	1.17 (1.12–1.22)***	1.16 (1.11–1.21)***
VAI Tertiles			
Q1	1 (Reference)	1 (Reference)	1 (Reference)
Q2	1.31 (1.14–1.51)***	1.30 (1.14–1.0)***	1.30 (1.13–1.49)***
Q3	1.63 (1.43–1.86)***	1.63 (1.43–1.86)***	1.62 (1.41–1.85)***
*P*_*interaction*_* for sex*	0.34	0.45	0.44

	Un-Adjusted	Multivariate Model 1	Multivariate Model 2
Body shape index (ABSI)	HR (95% CI)		
All (per 1-standard unit increment)	0.97 (0.91–1.03)	0.96 (0.91–1.02)	0.97 (0.91–1.03)
ABSI Tertiles			
Q1	1 (Reference)	1 (Reference)	1 (Reference)
Q2	1.03 (0.91–1.17)	1.03 (0.90–1.17)	1.03 (0.91–1.17)
Q3	0.94 (0.82–1.07)	0.93 (0.82–1.06)	0.94 (0.82–1.07)
*P*_*interaction*_* for sex*	0.40	0.53	0.61

HRs and 95% CI of the CVAI and ABSI. Model 1: Adjusted for hypertension (+ Age for VAI and ABSI); Model 2: Adjusted for hypertension, smoking, alcohol drinking, and exercise (+ Age for VAI and ABSI)

*ABSI* a body shape index; *CVAI* Chinese visceral adiposity index; *VAI* Visceral adiposity index

*Denotes *P* < 0.05, ** denotes *P* < 0.01, ***denotes *P* < 0.001

VAI had the Youden index values for identifying diabetes in both gender (0.12); the optimal VAI cut-off was 52.5 in men and 71.0 in women. Multivariable logistic regression models demonstrated that among both sexes, persons in higher quartiles of CVAI, VAI or ABSI were more likely to have diabetes mellitus compared to persons in the lowest quartile. (Additional file 1: Table 3), with the association between CVAI or VAI and baseline diabetes being more evident in women than in men (all *P*_interaction_ < 0.05). Overall, higher ABSI was also associated with higher diabetes mellitus risk, even after adjustment in both genders. These associations were, however, less prominent than those for CVAI or VAI (adjusted OR < 2 in models). Among the four anthropometric indices, CVAI had the highest area under ROC (AUC) for baseline diabetes in men (AUC = 0.65, 95% CI: 0.62–0.67), in women (AUC = 0.68, 95% CI: 0.66–0.70), and in all participants (AUC = 0.66, 95% CI: 0.65–0.68), followed by WC (AUC: 0.63, 0.66 and 0.65 for male, female, and all subjects, respectively) and BMI (Additional file 1: Fig. 2 A-C). VAI showed slightly attenuated discriminatory ability compared to CVAI in men (AUC = 0.63), with comparable ability in women (AUC = 0.68) (Additional file 1: Fig. 2). However, ABSI had the lowest AUC for diabetes in men (AUC = 0.56, 95% CI: 0.54–0.58), in women (AUC = 0.57, 95% CI: 0.55–0.59), and in all subjects (AUC = 0.57, 95% CI: 0.55–0.58). CVAI alone led to a significantly increased AUC over WC and other indices regardless of gender (all ∆ AUC *P* < 0.05, Additional file 1: Fig. 2 A-C). Additional file 1: Table 2 shows the sensitivity, specificity, and corresponding optimal cut-off values of each index for identifying diabetes by gender. The optimal CVAI cut-off for identifying baseline diabetes mellitus was 126.09 in men and 117.77 in women.

**Fig. 2:**
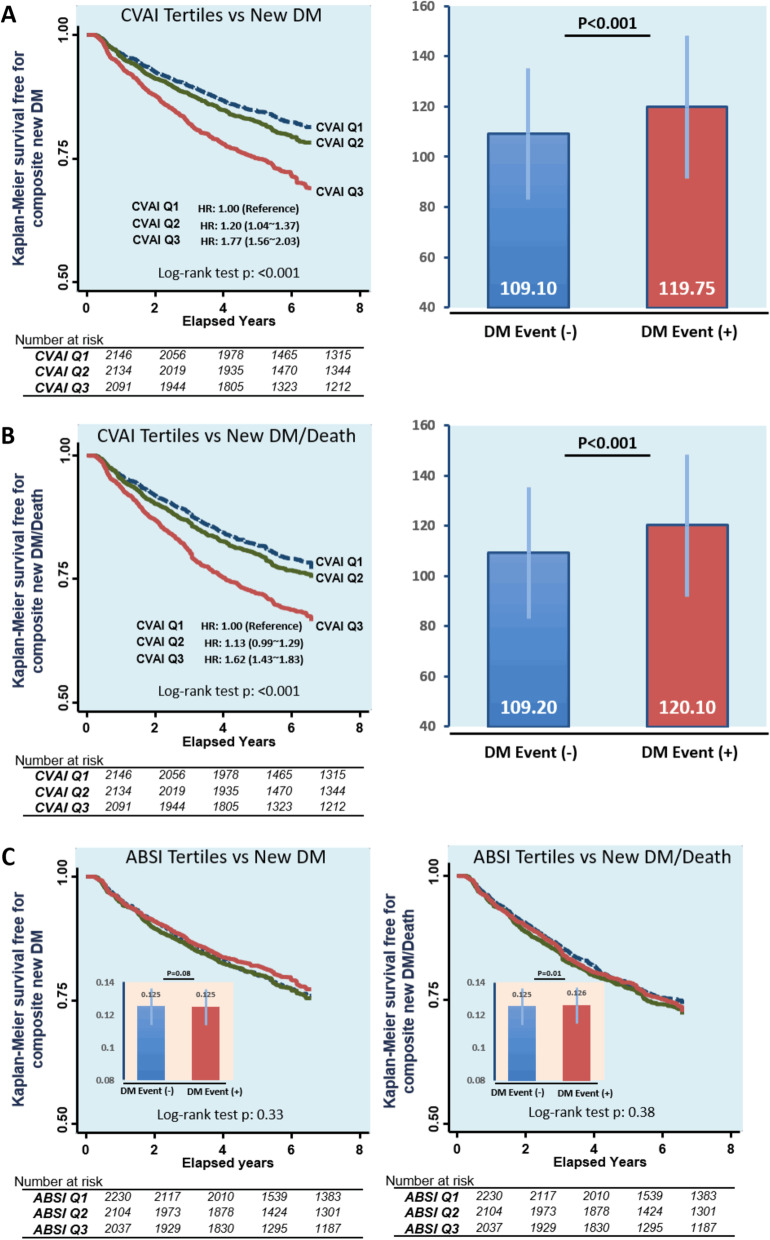
Kaplan–Meier survival curves of new-onset DM or a composite of new-onset DM/death across CVAI (**A**, **B**)/ABSI (**C**) tertile groups and difference of CVAI/ABSI between those who with or without events. *CVIA* Chinese visceral adiposity index; *ABSI* a Body Shape Index; *DM* diabetes mellitus

### Adiposity measures as a predictor of new-onset diabetes

Out of 6710 baseline non-diabetes mellitus subjects, 1360 developed new-onset type 2 diabetes mellitus during a median of 5.25 years (IQR: 3.07–6.44 years) follow-up. The number of mortality events recorded was 491, resulting in a combined 1699 subjects with new-onset diabetes mellitus or death. Higher prevalence rates of new-onset diabetes mellitus were observed across CVAI tertile groups (15.7%, 18.7%, and 26.4% for CVAI Q1, Q2, and Q3, respectively, *P* < 0.001) and new-onset diabetes mellitus/mortality group (19.0%, 21.5%, and 29.2% for CVAI Q1, Q2, and Q3, respectively, *P* < 0.001), with significantly higher CVAI observed in both cases (both *P* < 0.05) (Fig. 2). New onset of diabetes mellitus was not statistically different across ABSI tertiles (20%, 21.1%, and 18.9% for ABSI Q1, Q2, and Q3, respectively, *P* = 0.144), nor was composite new-onset diabetes mellitus and death (22.4%, 24.2%, and 23.0% for ABSI Q1, Q2, and Q3, respectively, *P* = 0.34) (Fig. 2). Both higher CVAI and VAI were independently predictive for new-onset diabetes mellitus (adjusted hazard ratio [aHR]: adjusted HR: 1.29, 95% CI: 1.22–1.37 and 1.16, 95% CI: 1.11–1.21 by CVAI and VAI) (Table 2) or composite new-onset diabetes mellitus/death (aHR: 1.23, 95% CI: 1.16–1.29 and 1.19, 95% CI: 1.13–1.25, both *P* < 0.001) (Additional file 1: Table 4) in adjusted models, although these associations were non-significant when ABSI was used.

## Discussion

This study assessed the associations of body adiposity indices, including CVAI and ABSI, with diabetes risk on a large scale in an older adult Chinese population. Our data showed that CVAI score strongly correlated with CT-defined visceral fat burden and was associated with several cardiometabolic risk profiles; however, the associations between these cardiometabolic risk profiles and ABSI were less prominent. While computed tomography (CT) remains the most commonly used imaging modality to measure abdominal fat [12, 13], magnetic resonance imaging (MRI) has a similar accuracy [22], to date, there is no clear definition and quantification of TAT using MRI measure. Overall, unlike ABSI, CVAI showed superior discriminatory abilities and could outperform conventional anthropometrics (such as BMI and WC) as a marker in identifying underlying diabetes mellitus in our older adult population, especially in women. Higher CVAI further showed independent predictive values for new onset diabetes mellitus and all-cause death during mid-term follow-up.

In line with previous reports, the CVAI or ABSI in our study showed positive correlations with visceral fat burden. All anthropometric indices were able to identify baseline diabetes mellitus (all AUC > 0.5). CVAI alone yielded the highest AUC (0.66) for diabetes among all anthropometric indices, followed by WC and BMI, and ABSI exhibited the weakest association with diabetes in both genders. Although several meta-analyses comprising multiethnic populations worldwide have shown that several anthropometric measures (including VAI, BMI, and WC) were strongly associated with diabetes risk [19, 23], CVAI as a marker of central adiposity has outperformed conventional anthropometric measures as a reliable diabetes mellitus marker in the Chinese population [14, 24] and was shown to successfully predict incident DM in ethnic Chinese [18, 19, 25] and other races [26, 27]. We further extended their findings to the older adult Chinese population. CVAI can therefore be a useful clinical adiposity surrogate for as a first-line screening tool (cut-offs of 126.09 and 117.77 for men and women, respectively) for diabetes mellitus in the older adult Chinese population when solid or more advanced biological specimens are not available. Given these associations, we postulated, by synergistically integrating information about BMI, central obesity along with lipid profiles (HDL and triglyceride), CVAI may better reflect adverse systemic effects from excessive visceral adiposity as a hallmark feature for insulin resistance and diabetes in Asians [28, 29].

ABSI was developed based on the United States National Health and Nutrition Examination Survey (NHANES) data (from 1999 to 2004), which provided useful health indicators from multiple ethnicities [14]. Previous reports demonstrating strong correlations between ABSI and metabolic disorders were mainly done in Caucasians [30, 31]. Given the metrics of height and weight, ABSI likely provides a reflection of VAT accumulation [32], although many subsequent studies showed that ABSI was neither clinically feasible nor valid in cardiometabolic comorbidity and mortality [32–34]. In agreement with our study results, the use of ABSI failed to provide additional values beyond conventional anthropometrics among older adult Indonesians in an earlier study [35] and also failed to differentiate between excess central adiposity, incidence of metabolic abnormalities or diabetes mellitus in multiple Asian regions [14, 32, 36–38]. As it has been proven, ethnic Asians are prone to metabolic abnormalities even at smaller WC and BMI measures when compared with Caucasians [39, 40]. This finding, along with the fact that there is a higher genetic predisposition of TG to act as an effective predictor of diabetes mellitus in Asians [41, 42], probably can explain the observed disparity between CVAI and ABSI, with the former serving as a useful marker for identifying diabetes.

Our study found that the new onset of DM nor combined new-onset diabetes mellitus and death was not statistically different across ABSI score levels. According to previous studies, Gujral et al. found a high prevalence of DM in normal-weight groups in nonwhite populations (racial/ethnic populations) [43]. In one prospective study, sponsored by the Indian Council of Medical Research (ICMR), they observed that about one-fourth of type 2 DM patients had a body mass index (BMI) below 19 kg/m^2^ (low body weight/lean body) [44]. From previous studies' conclusions, new or prevalent DM may not start with a very high BMI or body weight, and therefore an indicator that only relies on body size (such as ABSI) may fail to predict new DM in individuals of Chinese ethnicity.

Our study showed that the discriminative capability of CVAI in identifying diabetes mellitus was more prominent in older adult women than in men, which was not seen with ABSI. Our results also support the conclusion of Hameed et al. whose study focused on 300 type 2 DM women aged 25–60 years and found that VAI had a good predictive ability to identify the state of glycemic control as compared to other anthropometric measures (WC, BMI) or combined metabolic and anthropometric measures [45]. Visceral adiposity has been shown to be more sensitive and is a better indicator of insulin resistance and diabetes than BMI and WC, particularly in women [14, 46], and may serve as a prominent feature of metabolic abnormalities and diabetes. This may be related to the physiological differences between both genders in terms of visceral fat deposition and distribution and reproductive hormones [14, 46]. In addition, many studies found that VAI is not associated with DM among young participants (< 40 years) [46]. The impact of age can be explained by the Cartier et al. study that proposed that changes in the concentration of inflammation markers due to age is significantly related to the increase visceral adiposity distribution in the older adult. [47]

Though the precise reason behind some of these results remain to be clarified, this in part is hypothesized to be due to gender differences in visceral fat distribution [48] or due to the fact that women in Asia generally have greater abdominal adiposity and obesity, thus increasing related cardio-metabolic risks (i.e. HDL or TG) [9]. In this regard, CVAI can be particularly useful as an indicator of diabetes mellitus in the female population in Asia [24, 38]. To the best of our knowledge, this work represents the first large-scale study demonstrating the clinical usefulness of CVAI as a potential screening tool of diabetes in a large-scale older adult Chinese population. The collected data provides a wealth of relevant metrics that allow us to assess the predictive power of various associations; it also gives us a set of indicators for further assessments.

Our study has several limitations. Since the study was conducted in a single center, our observations were cross-sectional. Thus, these data cannot be used to make causal inferences regarding the relationships between CVAI, ABSI, and diabetes mellitus risks. Furthermore, we did not directly measure IR, and we were unable to assess the association of CVAI/ABSI with insulin resistance directly. Finally, data on postprandial glucose levels were not available, which can lead to the underdiagnoses of some subjects with diabetes mellitus.

## Conclusions

In conclusion, our study demonstrated stated that both CVAI and ABSI scores are strong and independent risk factors for diabetes mellitus among the older adult Taiwanese population. CVAI was found to be superior to WC, BMI, and ABSI and thus possesses the best predictive power for diabetes identification, based on the Youden index scores obtained in both genders (better scores in women compared with men). Our current analysis thus demonstrated that CVAI score, rather than ABSI, is better at identifying diabetes mellitus, when compared with BMI and WC measurements, in the older adult ethnic Chinese population, and it can also independently predict new-onset diabetes mellitus.

## Supplementary Information

**Additional file 1**. **Supplemental Figure 1.** Linear correlation between of MDCT-defined PCF/ TAT burden and CVAI and ABSI ABSI, a body shape index; CVAI, Chinese visceral adiposity index; PCF-peri-cardiac fat; TAT-peri-aortic fat. **Supplemental Figure 2.** The AUC for CVAI, ABSI, BMI, and WC for identifying baseline diabetes among elderly. **Supplemental Table 1.** Correlation of CVAI and ABSI with metabolic variables among elderly. **Supplemental Table 2.** Sensitivity, specificity, Youden index, and sex-specific cut-off points for various obesity indices in predicting DM risk among elderly. **Supplemental Table 3.** Uni- and multivariate models in identifying baseline diabetes risk by CVAI and ABSI in the current study population (n = 8249). **Supplemental Table 4.** Uni- and multivariate Cox models in predicting composite outcomes of new onset diabetes and death by CVAI and ABSI tertiles among study population without baseline diabetes (n = 6710).

## Data Availability

All data were fully anonymized.

## Abbreviations

ABSI: A body shape index
CT: Computed tomography
CVAI: Chinese visceral adiposity index
DBP: Diastolic blood pressure
eGFR: Estimated glomerular filtration rate
FPG: Fasting plasma glucose
HDL-C: High-density lipoprotein-cholesterol
HTN: Hypertension
LDL-C: Low-density lipoprotein cholesterol
MDCT: Multidetector of computed tomography
PCF: Peri-cardiac fat
ROC: Receiver operating characteristic
SBP: Systolic blood pressure
TAT: Peri-aortic fat
TC: Total cholesterol
TG: Triglycerides
UA: Uric acid
VAI: Visceral adiposity index
VAT: Visceral adipose tissue
WC: Waist circumference
BMI: Body mass index
