# Supplementary material for: Visceral adiposity index outperforms conventional anthropometric assessments as predictor of diabetes mellitus in elderly Chinese: a population-based study
Source: Nutr Metab (Lond). 2021 Sep 25;18:87. doi: 10.1186/s12986-021-00608-6 (PMC8465784; doi:10.1186/s12986-021-00608-6)
Supplement: Supplementary file 1 — Additional file 1. Supplemental Figure 1. Linear correlation between of MDCT-defined PCF/ TAT burden and CVAI and ABSI ABSI, a body shape index; CVAI, Chinese visceral adiposity index; PCF-peri-cardiac fat; TAT-peri-aortic fat. Supplemental Figure 2. The AUC for CVAI, ABSI, BMI, and WC for identifying baseline diabetes among elderly. Supplemental Table 1. Correlation of CVAI and ABSI with metabolic variables among elderly. Supplemental Table 2. Sensitivity, specificity, Youden index, and sex-specific cut-off points for various obesity indices in predicting DM risk among elderly. Supplemental Table 3. Uni- and multivariate models in identifying baseline diabetes risk by CVAI and ABSI in the current study population (n = 8249). Supplemental Table 4. Uni- and multivariate Cox models in predicting composite outcomes of new onset diabetes and death by CVAI and ABSI tertiles among study population without baseline diabetes (n = 6710). [file 12986_2021_608_MOESM1_ESM.docx]

**(Supplementary Materials)**

**Supplemental Figure 1**

**
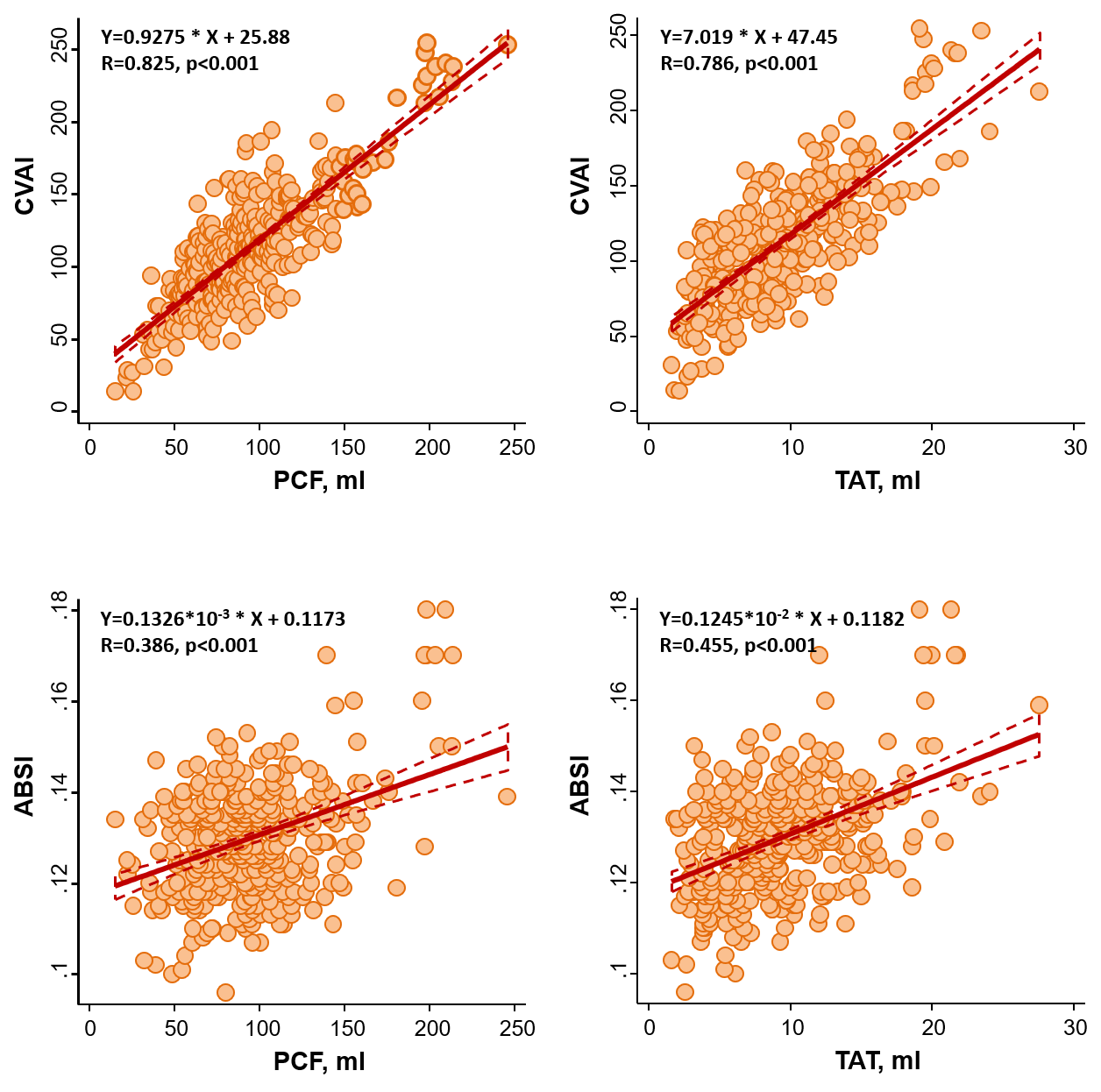
**

**Supplemental Figure 1**

Linear correlation between of MDCT-defined PCF/ TAT burden and CVAI and ABSI

ABSI, a body shape index; CVAI, Chinese visceral adiposity index; PCF-peri-cardiac fat; TAT-peri-aortic fat

**Supplemental Figure 2**

**
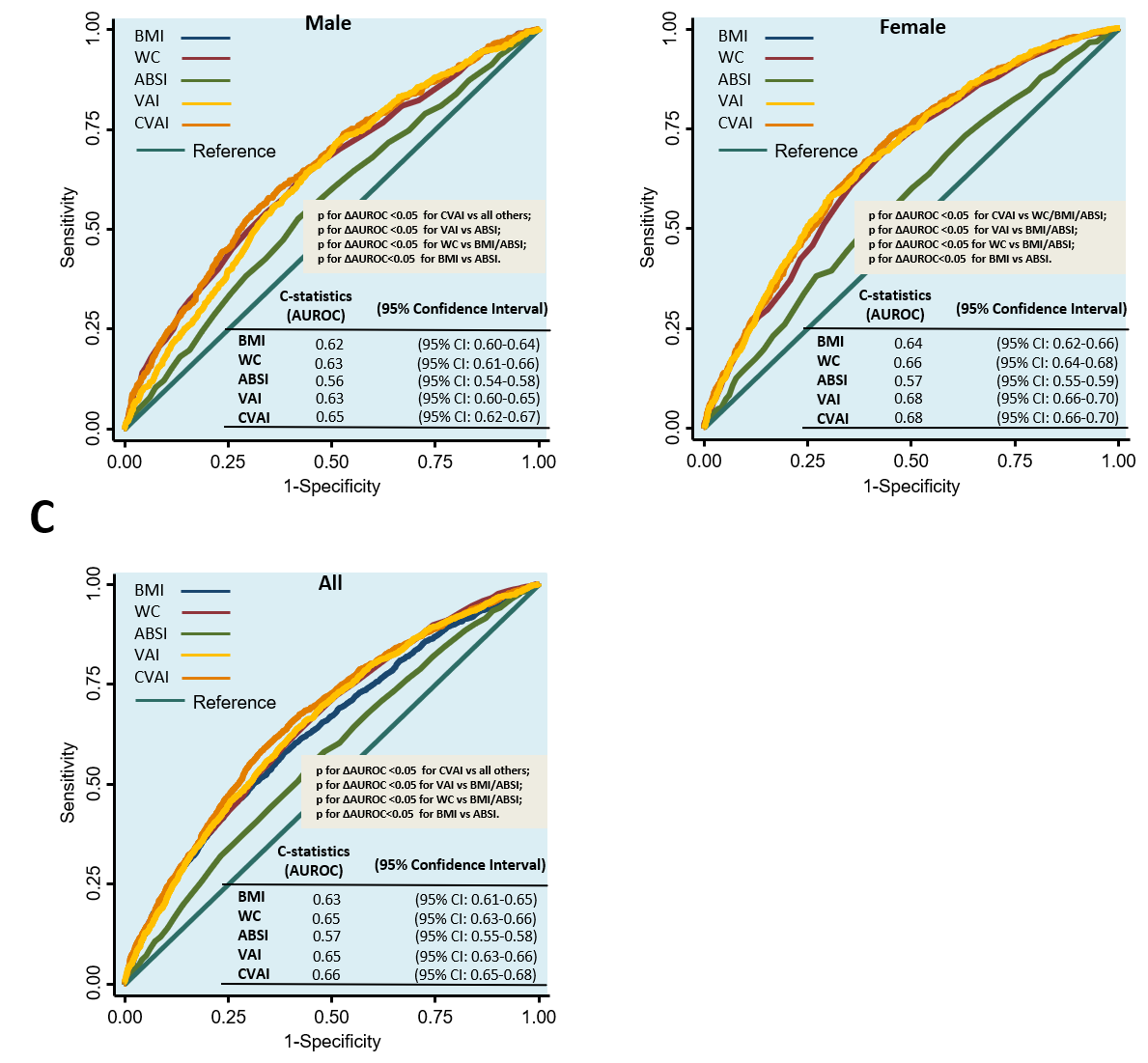
**

**Supplemental Figure 2**

The AUC for CVAI, ABSI, BMI, and WC for identifying baseline diabetes among elderly males (A), females (B), and among both genders (C). AUCs in identifying baseline DM were 0.65/0.63, 0.68/0.68, and 0.66/0.65 for CVAI/VAI in males, females, and all participants, respectively.

ABSI, a body shape index; AUC, area under the receiver operating characteristic curve; BMI, body mass index; CVAI, Chinese visceral adiposity index; WC, waist circumference; VAI, Visceral adiposity index.

**Supplemental Table 1.** Correlation of CVAI and ABSI with metabolic variables among elderly

|  | **Men** | | | | | |
| --- | --- | --- | --- | --- | --- | --- |
|  | **CVAI** | | **VAI** | | **ABSI** | |
|  | ***Pearson Correlation*** | ***P*** | ***Pearson Correlation*** | ***P*** | ***Pearson Correlation*** | ***P*** |
| BMI | 0.81 | < 0.001 | 0.37 | < 0.001 | 0.02 | 0.21 |
| SBP | 0.12 | < 0.001 | 0.07 | < 0.001 | -0.003 | 0.88 |
| DBP | 0.10 | < 0.001 | 0.08 | < 0.001 | 0.05 | 0.005 |
| WC | 0.97 | < 0.001 | 0.36 | < 0.001 | 0.55 | < 0.001 |
| HbA1c | 0.24 | <0.001 | 0.15 | < 0.001 | 0.14 | <0.001 |
| FPG | 0.22 | < 0.001 | 0.19 | < 0.001 | 0.11 | < 0.001 |
| TC | –0.09 | < 0.001 | 0.03 | 0.059 | –0.04 | 0.025 |
| TG | 0.37 | < 0.001 | 0.94 | < 0.001 | 0.08 | < 0.001 |
| HDL-C | –0.51 | < 0.001 | –0.57 | < 0.001 | –0.16 | < 0.001 |
| LDL-C | -0.03 | 0.004 | -0.12 | < 0.001 | -0.009 | 0.58 |
| UA | 0.22 | < 0.001 | 0.21 | < 0.001 | 0.06 | 0.003 |
|  | **Women** | | | | | |
|  | **CVAI** | | **VAI** | | **ABSI** | |
|  | ***Pearson Correlation*** | ***P*** | ***Pearson Correlation*** | ***P*** | ***Pearson Correlation*** | ***P*** |
| BMI | 0.81 | < 0.001 | 0.35 | < 0.001 | -0.12 | <0.001 |
| SBP | 0.24 | < 0.001 | 0.13 | 0.318 | -0.01 | 0.37 |
| DBP | 0.13 | < 0.001 | 0.14 | 0.63 | -0.005 | 0.75 |
| WC | 0.83 | < 0.001 | 0.34 | < 0.001 | 0.46 | < 0.001 |
| HbA1c | 0.30 | <0.001 | 0.27 | <0.001 | 0.12 | <0.001 |
| FPG | 0.25 | < 0.001 | 0.27 | < 0.001 | 0.06 | < 0.001 |
| TC | –0.09 | < 0.001 | 0.02 | 0.22 | –0.03 | 0.06 |
| TG | 0.48 | < 0.001 | 0.92 | < 0.001 | 0.08 | < 0.001 |
| HDL-C | –0.51 | < 0.001 | –0.62 | < 0.001 | –0.10 | < 0.001 |
| LDL-C | -0.04 | 0.006 | -0.06 | <0.001 | -0.02 | 0.26 |
| UA | 0.37 | < 0.001 | 0.27 | 0.001 | 0.04 | 0.017 |

BMI, body mass index; SBP, systolic blood pressure; DBP, diastolic blood pressure; FPG, fasting plasma glucose; WC, waist circumference; TC, total cholesterol; TG, Triglyceride; HDL-C, high density lipoprotein cholesterol; LDL-C, low density lipoprotein cholesterol; UA, uric acid.

**Supplemental Table 2.** Sensitivity, specificity, Youden index, and sex-specific cut-off points for various obesity indices in predicting DM risk among elderly.

|  | **Men (n = 3600)** | | | | **Women (n = 4649)** | | | |
| --- | --- | --- | --- | --- | --- | --- | --- | --- |
|  | **Cut-off** | **Sensitivity**  **(%)** | **Specificity**  **(%)** | **Youden**  **Index** | **Cut-off** | **Sensitivity**  **(%)** | **Specificity**  **(%)** | **Youden**  **Index** |
| CVAI | 126.09 | 53.60 | 69.46 | 0.23 | 117.77 | 73.19 | 54.80 | 0.28 |
| VAI | 52.5 | 57.3 | 54.4 | 0.12 | 71.0 | 55 | 56.8 | 0.12 |
| ABSI | 0.13 | 52.22 | 58.65 | 0.11 | 0.12 | 52.02 | 59.32 | 0.11 |
| BMI | 25.97 | 44.88 | 73.21 | 0.18 | 24.81 | 58.02 | 62.87 | 0.21 |
| WC | 89.95 | 53.46 | 66.92 | 0.20 | 81.40 | 68.79 | 56.92 | 0.26 |

ABSI, body shape index; CVAI, Chinese visceral adiposity index; DM, diabetes mellitus; BMI, body mass index; WC, waist circumference.

**Supplemental Table 3.** Uni- and multivariate models in identifying baseline diabetes risk by CVAI and ABSI in the current study population (n = 8249).

|  | **Unadjusted** | **Multivariate Model 1** | **Multivariate Model 2** |
| --- | --- | --- | --- |
| **Chinese Visceral adiposity index (CVAI)** | **OR (95% CI)** | | |
| **All Participants** |  |  |  |
| Q1 | 1 (Reference) | 1 (Reference) | 1 (Reference) |
| Q2 | 1.63 (1.34–1.98)*** | 1.68 (1.38–2.04)*** | 1.69 (1.38–2.06)*** |
| Q3 | 2.73 (2.27–3.28)*** | 2.91 (2.42–3.51)*** | 2.92 (2.42–3.52)*** |
| Q4 | 4.63 (3.88–5.53)*** | 4.95 (4.14–5.92)*** | 4.85 (4.05–5.82)*** |
| **Men** |  |  |  |
| Q1 | 1 (Reference) | 1 (Reference) | 1 (Reference) |
| Q2 | 1.44 (1.09–1.89)** | 1.45 (1.10–1.90)** | 1.44 (1.10–1.90)** |
| Q3 | 2.04 (1.57–2.65)*** | 2.11 (1.62–2.74)*** | 2.09 (1.61–2.72)*** |
| Q4 | 3.68 (2.87–4.72)*** | 3.76 (2.93–4.83)*** | 3.65 (2.84–4.69)*** |
| **Women** |  |  |  |
| Q1 | 1 (Reference) | 1 (Reference) | 1 (Reference) |
| Q2 | 1.87 (1.40–2.49)*** | 1.97 (1.48–2.63)*** | 2.02 (1.51–2.70)*** |
| Q3 | 3.61 (2.77–4.72)*** | 3.98 (3.04–5.21)*** | 4.06 (3.10–5.33)*** |
| Q4 | 5.85 (4.52–7.59)*** | 6.54 (5.04–8.50)*** | 6.53 (5.01–8.51)*** |
| *P _interaction_ for sex* | 0.008 | 0.003 | 0.002 |
| **Visceral adiposity index (VAI)** | **OR (95% CI)** | | |
| **All Participants** |  |  |  |
| Q1 | 1 (Reference) | 1 (Reference) | 1 (Reference) |
| Q2 | 1.77 (1.46–2.14)*** | 1.80 (1.49–2.18)*** | 1.78 (1.47–2.16)*** |
| Q3 | 2.53 (2.11–3.04)*** | 2.65 (2.20–3.19)*** | 2.61 (2.17–3.14)*** |
| Q4 | 4.17 (3.49–4.97)*** | 4.35 (3.64–5.19)*** | 4.22 (3.53–5.05)*** |
| **Men** |  |  |  |
| Q1 | 1 (Reference) | 1 (Reference) | 1 (Reference) |
| Q2 | 1.44 (1.09–1.89)** | 1.76 (1.38–2.25)*** | 1.73 (1.36–2.21)*** |
| Q3 | 2.04 (1.57–2.65)*** | 2.71 (2.14–3.44)*** | 2.65 (2.09–3.35)*** |
| Q4 | 3.68 (2.87–4.72)*** | 3.14 (2.45–4.02)*** | 3.00 (2.34–3.85)*** |
| **Women** |  |  |  |
| Q1 | 1 (Reference) | 1 (Reference) | 1 (Reference) |
| Q2 | 2.25 (1.62–3.13)*** | 2.25 (1.61–3.12)*** | 2.23 (1.61–3.11)*** |
| Q3 | 3.12 (2.27–4.28)*** | 3.26 (2.37–4.48)*** | 3.22 (2.34–4.43)*** |
| Q4 | 6.82 (5.05–9.20)*** | 7.02 (5.19–9.50)*** | 6.81 (5.03–9.22)*** |
| *P _interaction_ for sex* | <0.001 | <0.001 | <0.001 |
| **Body shape index (ABSI)** | **OR (95% CI)** | | |
| **All Participants** |  |  |  |
| Q1 | 1 (Reference) | 1 (Reference) | 1 (Reference) |
| Q2 | 1.17 (0.99–1.38) | 1.15 (0.97–1.36) | 1.14 (0.96–1.35) |
| Q3 | 1.41 (1.20–1.66)*** | 1.39 (1.18–1.64)*** | 1.37 (1.16–1.62)*** |
| Q4 | 1.81 (1.54–2.12)*** | 1.76 (1.50–2.07)*** | 1.71 (1.46–2.01)*** |
| **Men** |  |  |  |
| Q1 | 1 (Reference) | 1 (Reference) | 1 (Reference) |
| Q2 | 1.00 (0.78–1.28) | 1.00 (0.78–1.28) | 0.99 (0.78–1.28) |
| Q3 | 1.39 (1.10–1.76)** | 1.38 (1.09–1.75)** | 1.34 (1.06–1.70)* |
| Q4 | 1.63 (1.30–2.06)*** | 1.64 (1.30–2.06)*** | 1.57 (1.25–1.99)*** |
| **Women** |  |  |  |
| Q1 | 1 (Reference) | 1 (Reference) | 1 (Reference) |
| Q2 | 1.33 (1.06–1.68)* | 1.31 (1.04–1.65)* | 1.28 (1.02–1.62)* |
| Q3 | 1.42 (1.13–1.79)** | 1.41 (1.12–1.77)** | 1.39 (1.10–1.75)** |
| Q4 | 1.97 (1.59–2.45)*** | 1.88 (1.51–2.35)*** | 1.83 (1.47–2.29)*** |
| *P _interaction_ for sex* | 0.580 | 0.601 | 0.552 |

ORs and 95% CIs of the CVAI and ABSI. Model 1: Adjusted for hypertension (+Age for VAI and ABSI); Model 2: Adjusted for hypertension, smoking, alcohol drinking, and exercise (+Age for VAI and ABSI). ABSI, a body shape index; CVAI, Chinese visceral adiposity index; VAI: Visceral adiposity index.

**P* < 0.05; ***P* < 0.01; ****P* < 0.001.

**Supplemental Table 4.** Uni- and multivariate Cox models in predicting composite outcomes of new onset diabetes and death by CVAI and ABSI tertiles among study population without baseline diabetes (n = 6710).

|  | **Unadjusted** | **Multivariate Model 1** | **Multivariate Model 2** |
| --- | --- | --- | --- |
| **Chinese Visceral adiposity index (CVAI)** | **HRs (95% CI)** | | |
| **All (per 1-standard unit increment)** | 1.25 (1.19–1.32)*** | 1.24 (1.18–1.31)*** | 1.23 (1.16–1.29)*** |
| **CVAI Tertiles** |  |  |  |
| Q1 | 1 (Reference) | 1 (Reference) | 1 (Reference) |
| Q2 | 1.15 (1.01–1.31)* | 1.14 (1.01–1.30)* | 1.13 (0.99–1.28) |
| Q3 | 1.65 (1.46–1.86)*** | 1.62 (1.44–1.84)*** | 1.56 (1.38–1.76)*** |
| *P _interaction_ for sex* | 0.002 | 0.002 | 0.10 |
| **Visceral adiposity index (VAI)** | **HRs (95% CI)** | | |
| **All (per 1-standard unit increment)** | 1.24 (1.18–1.31)*** | 1.19 (1.13–1.26)*** | 1.19 (1.13–1.25)*** |
| **VAI Tertiles** |  |  |  |
| Q1 | 1 (Reference) | 1 (Reference) | 1 (Reference) |
| Q2 | 1.17 (1.03–1.33)* | 1.17 (1.03–1.33)* | 1.15 (1.01–1.31)* |
| Q3 | 1.39 (1.23–1.57)*** | 1.41 (1.03–1.33)*** | 1.38 (1.22–1.56)*** |
| *P _interaction_ for sex* | 0.14 | 0.38 | 0.36 |
| **Body shape index (ABSI)** | **HRs (95% CI)** | | |
| **All (per 1-standard unit increment)** | 1.03 (0.97–1.09) | 1.02 (0.97–1.08) | 1.01 (0.96–1.07) |
| **ABSI Tertiles** |  |  |  |
| Q1 | 1 (Reference) | 1 (Reference) | 1 (Reference) |
| Q2 | 1.09 (0.97–1.23) | 1.06 (0.94–1.20) | 1.08 (0.96–1.22) |
| Q3 | 1.03 (0.91–1.16) | 0.98 (0.87–1.11) | 0.99 (0.88–1.12) |
| *P _interaction_ for sex* | 0.09 | 0.12 | 0.18 |

HRs and 95% CI for the CVAI, VAI and ABSI.

Model 1: Adjusted for hypertension (+Age for VAI and ABSI); Model 2: Adjusted for hypertension, smoking, alcohol drinking, and exercise (+Age for VAI and ABSI). ABSI, a body shape index; CVAI, Chinese visceral adiposity index; VAI: Visceral adiposity index.

**P* < 0.05; ***P* < 0.01; ****P* < 0.001.
